# Natural Killer Cell-Based Therapies Targeting Cancer: Possible Strategies to Gain and Sustain Anti-Tumor Activity

**DOI:** 10.3389/fimmu.2015.00605

**Published:** 2015-11-30

**Authors:** Carin I. M. Dahlberg, Dhifaf Sarhan, Michael Chrobok, Adil D. Duru, Evren Alici

**Affiliations:** ^1^Cell Therapies Institute, Nova Southeastern University, Fort Lauderdale, FL, USA; ^2^Cell and Gene Therapy Group, Center for Hematology and Regenerative Medicine (HERM), Karolinska University Hospital Huddinge, NOVUM, Stockholm, Sweden; ^3^Oncology-Pathology, Cancer Center Karolinska, Karolinska Institutet, Stockholm, Sweden; ^4^Division of Hematology, Oncology and Transplantation, Masonic Cancer Research Center, University of Minnesota, Minnesota, MN, USA; ^5^Hematology Center, Karolinska University Hospital Huddinge, Stockholm, Sweden

**Keywords:** natural killer cells, adoptive cell therapy, immunotherapy, cancer, clinical trials, expansion, tumor microenvironment, genetic modifications

## Abstract

Natural killer (NK) cells were discovered 40 years ago, by their ability to recognize and kill tumor cells without the requirement of prior antigen exposure. Since then, NK cells have been seen as promising agents for cell-based cancer therapies. However, NK cells represent only a minor fraction of the human lymphocyte population. Their skewed phenotype and impaired functionality during cancer progression necessitates the development of clinical protocols to activate and expand to high numbers *ex vivo* to be able to infuse sufficient numbers of functional NK cells to the cancer patients. Initial NK cell-based clinical trials suggested that NK cell-infusion is safe and feasible with almost no NK cell-related toxicity, including graft-versus-host disease. Complete remission and increased disease-free survival is shown in a small number of patients with hematological malignances. Furthermore, successful adoptive NK cell-based therapies from haploidentical donors have been demonstrated. Disappointingly, only limited anti-tumor effects have been demonstrated following NK cell infusion in patients with solid tumors. While NK cells have great potential in targeting tumor cells, the efficiency of NK cell functions in the tumor microenvironment is yet unclear. The failure of immune surveillance may in part be due to sustained immunological pressure on tumor cells resulting in the development of tumor escape variants that are invisible to the immune system. Alternatively, this could be due to the complex network of immune-suppressive compartments in the tumor microenvironment, including myeloid-derived suppressor cells, tumor-associated macrophages, and regulatory T cells. Although the negative effect of the tumor microenvironment on NK cells can be transiently reverted by *ex vivo* expansion and long-term activation, the aforementioned NK cell/tumor microenvironment interactions upon reinfusion are not fully elucidated. Within this context, genetic modification of NK cells may provide new possibilities for developing effective cancer immunotherapies by improving NK cell responses and making them less susceptible to the tumor microenvironment. Within this review, we will discuss clinical trials using NK cells with a specific reflection on novel potential strategies, such as genetic modification of NK cells and complementary therapies aimed at improving the clinical outcome of NK cell-based immune therapies.

## Introduction

Natural killer (NK) cells are lymphocytes of the innate immune system. They are cytokine producing and have cytotoxic ability to kill both viral infected and tumor cells. Tumor-killing lymphocytes were first reported in 1968 by Hellström et al. (1). Kiessling and colleagues, in parallel with Ronald Herberman’s research laboratory, defined a novel lymphocyte population named NK cells that are able to target tumor cells in 1975 (2–5). Unlike T cells and B cells, NK cell recognition is not governed by high-resolution antigen specificity. Target cell recognition is mediated by the signals delivered through several activating and inhibitory receptors. The balance between activating and inhibitory signals decides the response of NK cells. When there is a mismatch between an inhibitory subgroup of killer immunoglobulin-like receptors (KIRs) on NK cells and self-human leukocyte antigen (HLA) class I proteins on the surface of target cells the NK cells can get activated due to lack of inhibitory signals leading to lysis of the host cell. This mismatch mediates alloreactivity and is the strategy behind the missing-self concept (6). KIRs can be divided into two haplotypes; the A haplotype with predominantly inhibitory KIRs plus only one activating KIR and the B haplotype containing inhibitory and activating receptors (7). During NK cell education, KIRs go through a random sequential acquisition process where they get functionally competent after they encounter self-MHC class I molecules. Consequently, mature NK cell function is inhibited by self-MHC class I and KIR interaction (8). When a NK cell confronts a target cell without expression of self-MHC class I molecules, the inhibitory signals are not active and the NK cell gets activated.

The majority of NK cells, as well as certain T cell subpopulations, may express the receptor family NKG2. One of the ligands for most NKG2 receptors is HLA-E, which is expressed on all nucleated cells. NKG2-family consists of seven members: NKG2A, B, C, D, E, F, and H in which NKG2A and B are inhibitory receptors. NK cells also express activation receptors on the surface, such as natural cytotoxicity receptors (NCRs), DNAM-1, and receptor members of the 2B4 family. NCRs, including NKp30, NKp44, and NKp46, are one of the main and initial groups of NK cell-activating receptors identified and they recognize viral ligands, heat shock-associated proteins, or tumor antigens (9). NK cells can also get activated by crosslinking of Fc receptor CD16 to target cell leading to antibody-dependent cellular cytotoxicity (ADCC) and lysis of the target cell (10, 11).

Natural killer cells perform their cytotoxic activity through granzyme B- and perforin-mediated apoptosis or by expression of death receptor ligands such as FasL and TNF-related apoptosis-inducing ligand (TRAIL). While the release of cytolytic granules is one of the essential cytotoxic responses, perforin deficient NK cells can still kill tumor cells through Fas-mediated apoptosis (12). Moreover, TRAIL-TRAILR mediated cytotoxicity also plays an important role in eliminating the target cells. Various tumor cells express TRAIL death receptors, which could be upregulated by proteasome inhibitors such as bortezomib (13). Additionally, immunomodulatory drugs (IMiDs) such as lenalidomide upregulates TRAIL expression on NK cells that potentially enhance the TRAIL-mediated elimination of tumor cells (14, 15).

Natural killer cells are derived from hematopoietic stem cells (HSC) in the bone marrow. The differentiation from HSC can be divided into five stages based on surface markers [detailed review in Ref. (16)]. The stages can be identified by the following surface markers, CD34, CD117, CD94, and CD16 among the Lin^−^ events, where stage 1 is CD34^+^CD117^−^CD94^−^CD16^−^. First at stage 2, the cells are able to respond to IL-15, which is necessary for NK cell development (17, 18). In the transition between stage 2 and 3, they lose their CD34 expression. At stage 4, the NK cells are CD56^bright^, produce IFNγ, and are capable of cytotoxic killing of K562 cells *in vitro* (19). NK cells in stage 5 are CD56^dim^ and express CD16.

The majority of human NK cells are CD14^−^CD19^−^CD3^−^CD56^+.^ While most of the CD56^+^ cells express lower levels of CD56 (~90% CD56^dim^), they are potent cytotoxic killers of target cells and secrete cytokines such as IFNγ. Approximately 10% of peripheral NK cells express high levels of CD56 (CD56^bright^), have low cytolytic activity, and have the capacity to produce high titers of immunoregulatory cytokines. The cell surface phenotypes of these two subpopulations also differ in respect to the receptors they express: the CD56^bright^ population expresses the inhibitory receptor NKG2A that could also be expressed on CD56^dim^ NK cells. While the CD56^dim^ population expresses FcγRIIIa (CD16a) as well as the inhibitory receptors KIRs (20).

## NK Cells in Cancer

Natural killer cells recognize tumor cells by the activating receptors like NCRs, which detect the altered expression of their ligands on the tumor cell surface. Additionally, downregulation or lack of MHC class I molecules on the cell surface of tumor cells can trigger NK cell activation since it diminishes the inhibitory signals transduced through KIR-MHC interactions. Moreover, since NK cells’ target recognition and activation are mainly through NCRs and missing-self, this engagement could induce upregulation of FasL on the NK cell surface leading to an alternative pathway inducing apoptosis in tumor cells. Nevertheless, both IL-2 stimulation and NK cell activation through NCRs also upregulate Fas on NK cells that may initiate regulation of the NK cell activation and expansion (21, 22).

Many tumors have gained methods to evade the surveillance by NK cells and other members of the immune system. For example, 16 of 18 patients with acute myeloid leukemia (AML) had reduced NCR surface expression compared to healthy donor NK cells, resulting in reduced cytotoxic capacity against target cells (23). Another way for tumor cells to escape recognition by NK cells is upregulation of the non-classical MHC class I molecule HLA-G, which dampens NK cell responses (24, 25). In numerous malignancies, there are also abnormalities found in the NK cell population. Examples of this include defective expression of activating receptors found in hepatocellular carcinoma (26), metastatic melanoma (27), AML (23), chronic lymphocytic leukemia (CLL) (28), and multiple myeloma (29, 30) or defective NK cell proliferation in metastatic renal cell carcinoma (31) and chronic myelogenous leukemia (CML) (32).

In renal cell carcinoma, infiltrating NK cells have, compared to peripheral blood NK cells, increased expression of NKG2A receptor contributing to decreased NK cell activity (33). NKG2D is a well-studied activating receptor on NK cells. Membrane-bound NKG2D ligand has a stimulatory effect on immunity, while soluble NKG2D ligands have the opposite effect on immune system leading to metastatic cancer progression (34). Patients with colorectal cancer have increased serum titers of the soluble NKG2D ligand, MHC class I chain-related protein A (sMICA), compared to healthy controls, leading to downmodulation of activating and cytokine receptors on the NK cells (35). A potential way to reduce the risk of soluble NKG2D ligand is to give the patients neutralizing antibody treatment. Clinical observations demonstrate that patients treated with cytotoxic T lymphocyte-associated antigen 4 (CTLA-4) antibody blockade have reduced sMICA in a close correlation with increased titers of autoantibodies against MICA (36). Interestingly, a new report from Deng et al. shows that the soluble high-affinity ligand MUL1 causes NK cell activation and stimulates tumor rejection in mice, instead of inhibition of NK cells as earlier reported (37).

The potential benefits of NK cell-based cancer immunotherapy products have led to the design of *in vitro* methods aiming to cultivate NK cells in cGMP conditions. Some of these methods have already been tested in clinical trials, which will be discussed later in this review.

## Clinical-Grade NK Cell Products

**Figure 1:**
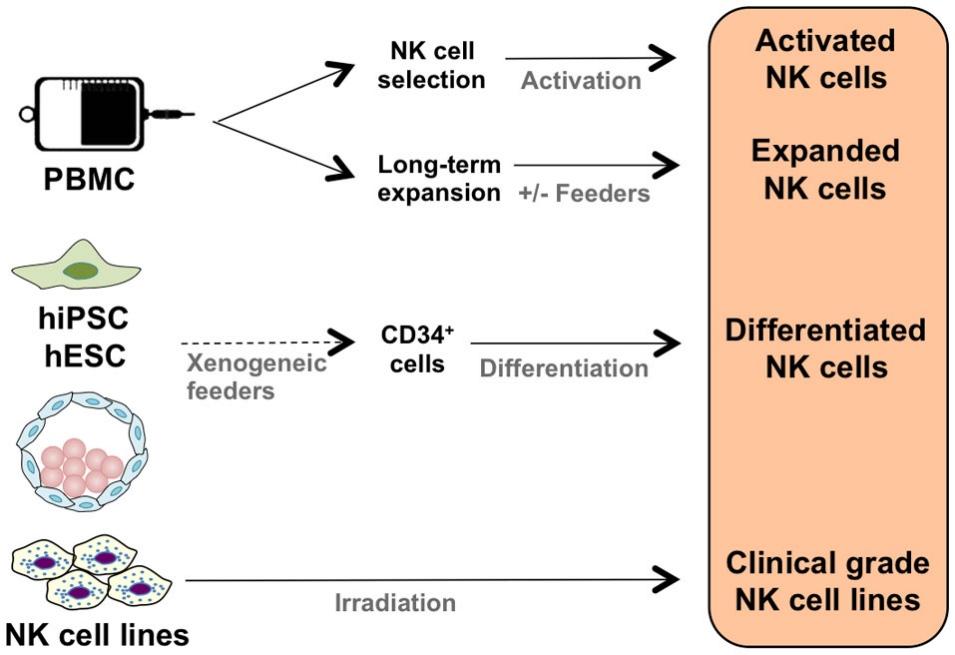
**Clinical NK cell therapy products**.

It is possible to activate NK cells and increase their anti-tumor activity through short-term cytokine exposure *in vitro* prior to adoptive transfer (38). However, to achieve clinically relevant numbers of NK cells, there also needs to be development of long-term NK cell expansion protocols (Table 1; Figure 1) (39–47). Yet, there are concerns when expanding NK cells *in vitro*, such as potential phenotypic changes, selective expansion, and reduced cytotoxic killing. When expanded *in vitro* with IL-2, there is a chance of CD3^+^ cell expansion as well (48, 49). Thus, there is still room for improvement to achieve optimum clinically relevant NK cell numbers, *in vivo* NK cell persistence and survival, and most importantly, anti-tumor activity. There are numerous parameters affecting the clinical-grade NK cell manufacturing such as source of the NK cells, cytokine stimulation, cell culture medium, and expansion platform. Here, in this section, we will address these parameters.

**Table 1:** **Clinical-grade NK cell products**.

Cell source	Medium	Serum	Feeder cell	Other	System	Time (days)	Purity (% NK cells)
UCB CD34+ cells (46)	GBGM	10% HS	–	High-dose cytokine cocktail (SCF, Flt3L, TPO, IL-7), low-dose cytokine cocktail (GM-CSF, G-CSF, IL-6), IL-15, low molecular weight heparin, high-dose cytokine cocktail (IL-7, SCF, IL-15, IL-2)	Vuelife bags	42	>90
					Wave bioreactor system		
					Biostat CultiBag system		
UCB CD34+ cells (50)	GBGM	2% HS	–	250 pg/mL G-CSF, 10 pg/mL GM-CSF, 50 pg/mL IL-6, high-dose cytokine cocktail (20 ng/mL IL-7, SCF, IL-15), 1000 U/mL IL-2, 200 pg/mL IL-12	Vuelife bags	21–28	>80
	SCGM						
NK-92 cell line (51)	X-Vivo 10, 15, 20	HS	–	450 IU/mL IL-2, 0.2 mM I-inositol, 2 mM L-glutamine, 20 mM folic acid, 10^−4^ M 2-mercaptoethanole	Flaske	15–17	–
	Aim VR	Human HP			Vuelife bags		
	TCM						
	QBSF-56	HSA			X-Fold culture bags		
NK-92 cell line (52)	X-Vivo 10	2.5% HP	–	500 U/mL IL-2, 0.6 mM l-asparagine, 3 mM l-glutamine, 1.8 mM l-serine	Vuelife culture bags	15–17	–
Total PBMC (48, 53)	CellGro SCGM	5% HS	–	500 U/mL IL-2, 10 ng/mL OKT3	Flasks	21	55–74
Total PBMC (54)	CellGro SCGM or RPMI-1640	10% FBS	K562-mb15-41BBL	10 U/mL IL-2	Flasks	7–14	96.8
					Teflon bags		83.1
Total PBMC (55)	RHAMα	5%AP	HFWT	100 U/mL IL-2	24-well plates	6–7	86
Total PBMC (42)	CellGro SCGM	5% HS	–	500 U/mL IL-2, 10 ng/mL OKT3	Flasks	20	65%
Total PBMC (45)	CellGro SCGM	5% HS	–	500 U/mL IL-2, 10 ng/mL OKT3	Wave bioreactor system	20	Relative: 64%
					Flasks		74%
					Vuelife bags		47%
Total PBMC (47)	GT-T510	1% HP	Autologous FN-CH296 induced T cells	IL-2, OK-432	CultiLife bag	20–21	90%
CD56 enriched PBMC (49)	X-VIVO 10	10% HS	–	500 U/mL IL-2, 10 ng/mL IL-15, 200 mM l-glutamine	NR	14	NR
CD5 and CD8 depleted PBMC (56)	RPMI-1640	10% HS	–	1000 U/mL IL-2, 2 mM l-glutamine, 1000 U/mL penicillin, 100 U/mL streptomycin	Polystyrene Cell Factories	21	88
					Teflon bags		
					Polyolefin bags		
CD5 and CD8 depleted PBMC (41)	2:1 DMEM:Ham’s F12-based NK medium	10% HS	–	1000 U/mL IL-2, 20 μM 2-mercaptoethanole, 50 μM ethanolamine, 20 mg/mL l-ascorbic acid, 5 μg/L sodium selenite, 100 U/mL penicillin, and streptomycin	Stirred-tank bioreactor	33	95–96
					Spinner flasks		
					24-well plates		
Non-adherent PBMC (57)	RPMI-1640	10% FBS	RPMI 8866	50 U/mL IL-2	24-well plates	10–12	80
Non-adherent PBMC (58)	RPMI-1640	10% FBS	RPMI 8866	50 U/mL IL-2	24-well plates	10–12	90
CD3 depleted non-adherent PBMC (39, 59)	DMEM	8% HS	LAZ 388	200 U/mL IL-2, 2 mM l-glutamine, 1 mM sodium pyruvate, 0.2% NaOH, 100 U/mL penicillin, 0.1 mg/mL streptomycin	V-bottom microplates	13–21	>90
Purified NK cells (60)	X-VIVO 20	–	Allogeneic mononuclear cells	100 U/mL IL-2, 10 U/mL IL-15, 100 μg/mL PHA, 1 μmol/mL ionomycin	Teflon bags	14–21	92
Purified NK cells (61)	X-VIVO 20	10% HS	EBV-TM-LCL	500 U/mL IL-2, 2 mM GlutaMAX-1 at 6.5% CO_2_	Flasks or Baxter bags	28	99
Adherent activated NK cells (40)	RPMI-1640	10% HS	Allogeneic mononuclear cells	6000 U/mL IL-2	Flasks	14–18	85

*PBMC, peripheral blood mononuclear cells; HS, human serum; FBS, fetal bovine serum; HP, human plasma; HAS, human serum albumin*.

### Source of the NK Cells

The majority of clinical NK cell products or pre-clinical research on efficient NK cell manufacturing platforms are making use of peripheral blood mononuclear cells (PBMC), umbilical cord blood (UCB), cell lines, and human embryonic stem cells (hESC), as well as induced pluripotent stem cells (iPSC) as a source of start material.

#### Peripheral Blood Mononuclear Cells

The majority of NK cell products are generated through utilization of PBMCs either by apheresis or ficoll separation under cGMP conditions. An advantage of using PBMCs is the ability to collect cells in a closed aseptic system. Although PBMC consists of 5–20% NK cells, it is not possible to achieve sufficient numbers of potent NK cells. Thus, various techniques to expand NK cells *ex vivo* have been developed. For example, we have designed a feeder-free NK cell expansion system where it is possible to expand and activate tumor-reactive NK cells in a clinically compatible manner (45). These cells have a high cytotoxic effect specifically against autologous and allogeneic tumors *in vitro* and *in vivo* (42, 45). We have also completed a first-in-man clinical trial using donor-derived *ex vivo* expanded NK cells in terminal cancer patients that had CLL, kidney cancer, colorectal cancer, and hepatocellular carcinoma with promising results (43). Having optimized the procedure for NK cell expansion in a closed-automated bioreactor using clinical-grade GMP-compliant components, we have initiated a first-in-man phase I/II clinical trial to expand and restore the function of patients’ own NK cells (45, 62). To our knowledge, this is the first advanced therapy investigational medicinal product trial performed using autologous NK cells in Sweden.

Sakamoto et al. have established another similar approach that generates large numbers of activated NK cells from peripheral blood without prior purification of the cells. The PBMCs are cultured with autologous plasma, IL-2, OK-432, and γ-irradiated autologous FN-CH296 stimulated T cells, reaching up to a median purity of 90.96% of NK cells at day 21 or 22. Many of the NK expansion protocols are based on enrichment of NK cells either prior to NK cell activation and expansion through cell selection or sorting in order to achieve pure cell therapy product and avoid unwanted side effects stemming from T cells especially in allogeneic NK cell transfusions.

One of the main methods of enriching the purity and the number of initial NK cells is the clinical-grade immuno-magnetic depletion of other lymphocyte subsets such as T cells and/or B cells as well as myeloid cells (60). Depletion of CD3^+^ cells followed by CD56^+^ cell enrichment can lead to highly pure NK cells which could be supplemented by CD19^+^ cell depletion before infusion in order to prevent passenger lymphocyte syndrome in allogeneic transplantation (63). Nguyen et al. have shown that a partial depletion of T cells could get a more beneficial clinical outcome compared to a complete T cell depletion after hematopoietic stem cell transplantation, suggesting that T cells may have a positive role in *in vivo* NK cell function (64).

Additionally, direct enrichment of CD56^+^ NK cells through immuno-magnetic selection is an option to achieve high purity initial NK cell product. Nevertheless, NK cells might require physical and cytokine-dependent communication with other cells such as monocytes (65) in order to activate and expand. Thus, it is essential to fine-tune the enrichment of NK cells by making use of feeder cells and/or optimizing the cytokine cocktail used in *ex vivo* NK cell expansion protocols.

Furthermore, using feeder cells and cell lines is another approach in expanding NK cells *ex vivo* since feeder cells can provide essential stimulatory signals for NK cells proliferation. Monocytes, irradiated PBMC, feeder cell lines, and engineered feeder cell lines are the most commonly used sources for stimulation of NK cell expansion through humoral signals and cell-to-cell contact. Example of feeder cells that have been used in clinical trials are irradiated autologous PBMCs (60, 66), irradiated Epstein–Barr virus-transformed lymphoblastoid cells (61), and K562 engineered cells expressing 4-1BB ligand (67) or membrane-bound IL-21 (68, 69) on cell surface.

#### Stem Cells

While PBMC is one of the major sources for achieving clinically relevant doses of tumor-reactive NK cells, HSC and potentially hESC as well as iPSC are likewise essential sources for achieving clinically relevant doses of NK cells.

One of the potential sources to accomplish clinically relevant doses of tumor-reactive NK cells is making use of HSC (CD34^+^) through differentiation and expansion of CD34^+^ cells isolated from bone marrow, peripheral blood, or UCB into functional NK cells. It was recently demonstrated that it is possible to expand activated, tumor cytotoxic and pure NK cells by differentiating UCB CD34^+^ HSC under cGMP condition (46). Furthermore, NK cells derived from CD34^+^ UCB cells lack expression of KIRs such as KIR2DL1 (CD158a), KIR2DL2/DL3 (CD158b), and NKB1, as well as diminished CD16 expression in the CD56^dim^ population (70). Even though NK cells derived from UCB have reduced cytotoxicity, this could be restored by *ex vivo* cytokine stimulation such as IL-2, IL-12, and IL-15 (50, 71–73). Infusion of UCB-derived NK cells supplemented with IL-15 has shown to inhibit growth of human bone marrow resident leukemia cells *in vivo* (74). Recently, it was demonstrated that frozen UCB CD34^+^ cells differentiate into NK cells with better expansion than freshly isolated UCB CD34^+^ cells, and more importantly, UCB CD34^+^ cells gave more NK cell product than peripheral blood HSC without jeopardizing NK cell functionality (75). Thus, UCB CD34^+^ cells are one of the essential sources for manufacturing NK cell therapy protocols, providing an option to create NK cell biobanks.

Another potential source of NK cells is hESC and iPSC, with the advantage of potential usage of iPSCs in autologous settings with reduced risk of immune rejection. The first step is to generate CD34^+^ hematopoietic precursor cells from the hESCs and iPSCs and then differentiate these cells into NK cells, which could be efficiently achieved through growing hESCs and iPSCs on murine stromal cells (76, 77). Yet, the involvement of xenogeneic cells could limit the potential clinical usage of hESCs and iPSCs. Addressing this potential problem, Knorr et al. developed a two-stage culture method where hESCs and iPSCs are first differentiated to CD34^+^ hematopoietic cells by spin-EB system in xeno-free and serum-free conditions followed by stroma-free NK cell differentiation, which enables generation of cytotoxic NK cells without involvement of xenogeneic cells taking a step forward toward clinical-scale production (78). Since IL-2-activated NK cells are potent killers of both allogeneic and autologous iPSCs (79), it is possible to manufacture a pure NK cell therapy product. This sticks out as one of the advantages of using *in vitro* NK cell differentiation from iPSCs.

#### Cell Lines

Cell lines derived from NK cells with similar biological functions (NK-92, NKL, KYHG-1, and NKG) are potential candidates for NK cell-based products enabling design and development of off-the-shelf anti-cancer cell therapy products. Furthermore, it is more feasible to generate genetically modified NK cell lines expressing intracellular IL-2 for activation or cell surface molecules such as CD16, NCRs, and chimeric antigen receptors (CARs). To our knowledge, the NK-92 cell line is the most clinically studied one. The IL-2-dependent NK-92 cell line is cytotoxic to a wide range of malignant cells (80–83). It has also been used as a source of NK cells for cGMP-grade cellular therapy products (51) as well as in clinical trials (52, 84). The NK-92 cell line expresses several activating receptors but lacks most of the inhibitory KIRs, NKp44, and CD16 (80, 85). NK-92 cells require irradiation to prevent proliferation prior to being used effectively in immunotherapeutic approaches without compromising hematopoietic cell function. For example, recently, clinical-grade NK-92 cells have been manufactured and were safely used as anti-tumor therapy for patients with a variety of tumors (84) with promising results (52). As of today’s date, two phase I clinical trials (NCT00900809 and NCT00990717) are recruiting patients with hematological malignancies for treatment with NK-92 cells. The first clinical phase II study (NCT02465957) with NK-92 cells has recently been initiated.

KHYG-1 is the first NK cell line derived from NK leukemia and has higher cytotoxicity than NK-92 cell line (86). Likewise NK-92 cells, these cells can also be irradiated to inhibit proliferation and can still efficiently kill tumor targets. Furthermore, NKL cell line, which is the most biologically and functionally similar to primary NK cells, is more cytotoxic to certain tumor cells than NK-92 cell line and, additionally, it has the ADCC capacity whereas NK-92 cells lack CD16 expression. Thus, both KHYG-1 and NKL cell lines have the potential to be used as anti-cancer NK cell products.

Additionally, one of the advantages of using such master cell bank is an appealing opportunity in the manufacture of cellular therapy products since it is possible to establish a comprehensive standardization and characterization of the cell source. It is also possible to genetically modify these cell lines to exert more tumor specificity and cytotoxicity. For example, NK-92 cell lines are dependent on external IL-2 stimulation, which increases manufacturing costs as well as potentially reducing the long-term cytotoxic capacity of these cells unless they are supported by IL-2 infusions. Thus, constitutive expression of IL-2 in NK-92 cells through genetic modification leads to auto-activated and -proliferating cells, which reduces the manufacturing costs as well as potentially increases the *in vivo* tumor reactivity (87, 88).

### Cytokines

*Ex vivo* manufacturing of NK cell-based products is dependent on extensive use of cytokines to stimulate, differentiate, activate, and expand NK cells in order to get clinically relevant doses and enhanced anti-tumor reactivity. Historically, one of the most popular cytokines in NK cell research is IL-2 since it was the first cytokine to be injected to patients to treat metastatic melanoma (89). Thirty years ago, Rosenberg et al. published the first report where they treated 25 metastatic cancer patients, who did not respond to standard therapy, with autologous lymphokine-activated killer (LAK) cells together with recombinant-derived IL-2. LAK cells are generated from mononuclear cells collected from IL-2 injected patients. In 11 patients, the cancer regression was observed with >50% of tumor volume (90). This adoptive immunotherapy was followed by a larger scale study, where 157 patients with advanced metastatic cancer were treated with successful results (91). In the same year, it was shown that it was the NK cells that mediated the cytotoxic activity in response to systemic administered recombinant IL-2 (92). These reports were followed by many years of IL-2 and NK cell research. In a dose-dependent manner, IL-2 is important for NK cell infiltration and killing of the tumor. For example, in the bone marrow, there are hypoxic regions leading to reduced NK cell killing of plasma cells in multiple myeloma. IL-2-activated NK cells *ex vivo* have increased NKG2D expression resulting in increased targeting of multiple myeloma upon infusion (93). Cytokine-activated NK cells *in vitro* are dependent on constant stimulation both *in vitro* and *in vivo*. Basse et al. reported that when no exogenous IL-2 is present the amount of injected NK cells found in tumors were very low (94). The half-life of IL-2 in serum is not more than 10 min, which makes the administration of IL-2-dependent cells difficult (95). By transducing NK cells to produce IL-2 prior to transplantation, the activated NK cells would have a constant source of IL-2 *in vivo* (87, 96). One of the disadvantages of using IL-2 to activate NK cell *in vivo* is the competition over IL-2 by regulatory T cells, which express high levels of the high-affinity receptor for IL-2, IL-2Rα (CD25). By treating patients with lympho-depleting agents (fludarabine and cyclophosphamide) followed by NK cell infusion and IL-2 fused with diphtheria toxin (IL-2DT), CD25^+^ cells are selectively depleted, leading to increased NK cell expansion and complete remission rate for patients with AML compared to regular IL-2 treatment (97). Overall, the majority of cGMP-grade NK cell therapy protocols include IL-2 as a main cytokine to stimulate NK cell activation and proliferation.

Another important cytokine is IL-15 which is required for both NK cell maturation and survival (98). IL-2 and IL-15 share the same receptor components: IL-2/15Rβ and common γ chain (also shared with IL-4, IL-7, IL-9, and IL-21). Recent advances in the production of cGMP quality cytokines enabled further optimization of cytokine supplementation during NK cell expansion. For example, use of IL-15 in combination with IL-2 has a synergetic effect on product viability and NK cell proliferation (66). This highlights the necessity of other cytokines to achieve NK cell product potency especially when it comes to the NK cell expansion protocols that are not using feeder cell support. Additionally, IL-21, primarily described in 2000 (99), has significant homology with IL-2 and IL-15. Compared to IL-2 and IL-15, IL-21 promotes maturation and survival but does not promote proliferation of NK cells alone. However, IL-21 does have synergetic effects with IL-2 and IL-15 (100). Interestingly, it has been suggested that IL-21 does not drive proliferation of regulatory T cells *in vivo* and might be a good candidate to substitute for IL-2 in CLL (101).

### Other Factors

Besides NK cell source, feeder support, and cytokine stimulation, other parameters such as expansion platform, cell culture media, and serum supplementation are also very important in achieving clinically relevant cell numbers, viability, and tumor cytotoxicity. More specifically, we have recently investigated the importance of the culture vessels on the quality and efficacy of the NK cell product. Briefly, PBMCs from healthy donors and myeloma patients were cultured for 21 days using flasks, cell culture bags, and bioreactors. Even though we have achieved high yield in NK cell expansions in all systems, NK cells expanded in the bioreactor displayed significantly higher cytotoxic capacity. These results demonstrate that highly active NK cells can be produced in a closed, automated, large-scale bioreactor under feeder-free current GMP conditions facilitating adoptive immunotherapy clinical trials (45).

Additionally, cell culture media is another important factor to consider in the manufacturing of cellular therapy products. There are very few cGMP quality medias that work optimally for *ex vivo* NK cell expansion protocols. The most commonly preferred media in the generation of NK cell products are stem cell growth medium (SCGM; CellGenix, Freiburg, Germany), X-VIVO serum-free media (BioWhittaker, Verviers, Belgium), or AIM V (Life Technologies, Grand Island, NY, USA) (49, 102, 103). Generally, medium is supplemented by human AB serum or fetal bovine serum from certified sources.

Finally, there are numerous variables that may impact quality and quantity of NK cell products. Future pre-clinical research and results from more clinical trials will evaluate the contribution of each factor to the product purity, potency, and safety, as well as assist in acquiring NK cell products that can be manufactured reproducibly with the optimal safety and anti-tumor responses.

## Clinical Use of NK Cell-Based Anticancer Products

### Autologous NK Cells

Several clinical studies have been performed with adoptive autologous NK cells in an attempt to target tumors, such as breast cancer, lymphoma, glioma renal cell carcinoma, non-small cell lung cancer, and adenocarcinoma (Table 2) (39, 40, 55, 103–107). In general, autologous NK cell trials are safe with no toxic side effects (39, 40, 55, 105). For example, *ex vivo* activated autologous peripheral blood lymphocytes get enhanced cytolytic activity against heat shock protein 70 (Hsp70) membrane-positive tumors *in vivo* if pre-incubated with Hsp70 peptide and IL-2 (105, 107). However, some clinical trials with autologous NK cells have only partial effect on tumors, such as glioma (55). While other tumors, such as metastatic carcinoma or relapsed lymphoma, do not demonstrate any improvement (103, 104, 108). Moreover, a recent clinical trial used *ex vivo* FN-CH296 stimulated T cells and OK-432 expanded, autologous NK cells with enrolled patients diagnosed with rectal, esophageal, gastric, or colon cancer that was either recurrent or at metastatic disease stage. The NK cell therapy in these patients was well tolerated with no severe adverse events and the cytotoxicity of peripheral blood was elevated approximately twofold up to 4 weeks post the last transfer (47).

**Table 2:** **Clinical trials with infusion of autologous NK cells**.

Malignancy	n	NK cell source	Depletion	Product	*Ex vivo* handling	Purity	Dose	Outcome
Colorectal carcinoma/NSCLC (105)	11/1	PBMC	–	IL-2 + Hsp70 peptide	4 days	Mean: 14% (range: 8–20%)	Range: 0.1–1.5 × 10^9^ NK cells	Cytotoxic activity of NK cells. No significant tumor response
Colon carcinoma (107)	1	PBMC	–	IL-2 + Hsp70 peptide	4 days	Mean: 22.4% (range: 16–25%)	Mean: 1.48 × 10^9^ NK cells (range: 0.9–1.9 × 10^9^)	Anti-tumor activity by NK cells
Glioma (55)	9	PBMC	–	Irradiated feeder cell line (HFWT) + autologous plasma + IL-2	14 days	82.2 ± 10.5%	i.c. 0.4–2.3 × 10^9^ cells	3 partial responses, 2 minimal responses
							i.v. 0.2–6.5 × 10^9^ cells	
RCC (39)	10	PBMC	CD3+ depletion or Immunorosette depletion	Cultured on LAZ388 with allogeneic irradiated PBMNC as feeder cells + IL-2	13–21 days	>90% except 1 patient (33%)	Mean: 5.8 × 10^9^ total cells (range: 1.8–15.1 × 10^9^)	All patients improved, 4 complete response, 2 partial response
Melanoma/RCC (103)	7/1	PBMC	CD3+ depletion	Autologous irradiated PBMNC as feeder cells + IL-2 and OKT3	21 days	96% ± 2%	Range: 4.7 × 10^10^ (±2.1 × 10^10^) NK cells	No tumor lysis by NK cells. No tumor response
Rectal/esophageal/gastric/colon cancer (47)	4/4/3/3	PBMC	–	Autologous FN-CH296 stimulated T cells + autologous plasma + IL-2 and OK-432	21–22 days	Median: 90.96% (range: 65.94 −99.45%)	0.5–2.0 × 10^9^ cells	No tumor response
Lymphoma/breast cancer (104)	20/14	*In vivo* IL-2 activated NK cell	–	IL-2	Over night	Not reported	Range: 0.33–2.09 × 10^8^ cells/kg	No improvement of survival
Breast cancer (108)	5	*In vivo* IL-2 activated NK cell	Monocyte depletion	Allogeneic irradiated PBMNC as feeder cells + IL-2	14 days	Mean: 83.2% (range: 67–93%)	Mean: 3.97 × 10^9^ total cells (range: 1.55–9.1 × 10^9^)	1 complete response, 1 partial response, 2 had stable disease, 1 disease progression
Lymphoma/breast cancer (40)	10/1	*In vivo* IL-2 activated NK cell progenitors	Monocyte depletion	Allogeneic irradiated PBMNC as feeder cells + IL-2	14–18 days	Mean: 85% (range: 64–98%)	Range: 6.8 × 10^8^–4 × 10^10^ total cells	Increased NK cell numbers and activity in 4 patients

*NSCLC, non-small cell lung cancer; PBMC, peripheral blood mononuclear cell; RCC, renal cell carcinoma*.

### Allogeneic NK Cells

Allogeneic NK cell products have been used in the treatment of a range of malignancies, such as leukemia, renal cell carcinoma, leukemia, colorectal cancer, hepatocellular cancer, lymphoma, and melanoma (Table 3) (38, 109–113). The major risk with allogeneic NK cell transplantation is the development of graft-versus-host disease (GvHD). Several precautions can be taken to reduce the risk of GvHD, for example, immunosuppression, infusion of CD3 depleted high purity NK cells and if available, selecting the donor that matches the host HLA (44, 114, 115).

**Table 3:** **Clinical trials with infusion of allogeneic NK cells**.

Malignancy	n	NK cell source	Depletion	Product	*Ex vivo* handling	Purity	Dose	Outcome
		**Cell line**						
RCC/MM (52)	11/1	NK-92	–	IL-2	3 weeks	Clonal cell line	1 × 10^8^–3 × 10^9^ cells/kg	1 mixed response, 4 stable disease, 6 progressive disease
Solid tumor/CLL/B-NHL (84)	13/1/1	NK-92	–	IL-2	2–2.5 weeks	Clonal cell line	1 × 10^9^, 3 × 10^9^, 1 × 10^10^ cells/m^2^	2 mixed response, 1 stable disease, 12 progressive disease
		**Progenitor cells**						
AML/ALL/high-grade MDS (114)	11/1/2	Related CD34+ progenitors	CD34+ selection	IL-15, IL-21+ hydrocortisone	42 days	Not reported	Mean: 3.49 × 10^8^ NK cells/kg (range: 1.8–6.3 × 10^8^)	2 with active leukemia had no response
		**Adult cells**						
AML/CML (110)	4/1	Haploidentical PBMC	CD3+ depleted, CD56+ enrichment	–	Overnight storage in +4°C	Median: 97.35% (range: 77.9–98.9%)	Median: 0.93 × 10^7^ cells/kg (range: 0.21–1.41 × 10^7^)	3 donor chimerism, 1 relapse
AML (112)	10	Haploidentical PBMC	CD3+ depleted, CD56+ enrichment	–	Overnight storage	Not stated	Mean: 29 × 10^6^ NK cells/kg (range: 5–81 × 10^6^)	*In vivo* expansion of NK cells. 2 years event-free remission in 100%
AML (113)	13	Haploidentical PBMC	CD3+ depleted, CD56+ enrichment	–	–	Median: 93.5% (range: 66.4–99.2%)	Median: 2.74 × 10^6^ cells/kg (range: 1.11–5 × 10^6^)	3 disease-free. 4 complete remissions, 5 with active disease had no clinical benefit
AML (116)	1	Haploidentical PBMC	CD3+ depleted	–	–	Not stated	3 × 10^7^ NK cells/kg	Complete response, relapse on day 80
Melanoma/RCC/HD/AML (38)	10/13/1/19	Haploidentical PBMC	CD3+ depleted	IL-2	Over night	Mean: 40% (range: 18–68%)	1 × 10^5^–2 × 10^7^ cells/kg	*In vivo* expansion of NK cells. 5 complete remission (AML)
Breast/ovarian carcinoma (115)	6/14	Haploidentical PBMC	CD3+ depleted	IL-2	Overnight	25.0 ± 0.3%	Mean: 2.15 × 10^7^ NK cells/kg	4 partial responses, 12 and 3 stable or progressive diseases, respectively
							8.33 × 10^6^–3.94 × 10^7^ cells	
Neurobalstoma/AML/ALL/RMS/HD (117)	4/5/5/1/1	Haploidentical PBMC	CD3+ depleted, CD56+ enrichment	Group 1: –	9–14 days	Median: 95% (range: 84.4–98.6%)	Range Group 1: 3.2–38.3 × 10^6^ cells/kg, Range Group 2: 6–45.1 × 10^6^ cells/kg	Group 1: 3 complete remissions (1 NB, 2 ALL)
				Group 2: IL-2				Group 2: 2 complete remissions (NB)
ALL/AML (109)	2/1	Haploidentical PBMC	CD3+ depleted, CD56+ enrichment	IL-2	14 days	Mean: 95%	Mean: 11.9 × 10^6^ cells/kg (range: 3.3–29.5 × 10^6^)	3 complete remissions, AML patient got early relapse
Neuroblastoma (118)	2	Haploidentical PBMC	CD3+ depleted, CD56+ enrichment	IL-2	14 days	>95%	7.8–45.1 × 10^6^ cells/kg	Initially enhanced NK cells cytotoxicity
CRC/HCC/RCC/CLL (43)	1/1/2/1	Haploidentical PBMC	–	IL-2 + OKT3	20 days	Not stated	1.0–10 × 10^6^ NK cells/kg	Signs of response in HCC. No tumor response
NSCLC (44)	15	Haploidentical PBMC	CD56+ enrichment	IL-15 + hydrocortisone	20–23 days	Median: 97.9% (range: 82.7–899.6%)	Median 4.15 × 10^6^ NK cells/kg (range: 0.2–29 × 10^6)^	15 months median overall survival. 56% 1-year-survival, 19% 2-year survival
MM (119)	8	Haploidentical PBMC (3) or autologous PBMC (5)	CD3+ depleted	IL-2 + irradiated K562-mb15-41BBL	7–9 days	Median: 78% (range: 52–90%)	2 × 10^7^–1 × 10^8^ NK cells/kg	1 partial response, 1 reduced disease progression, 5 no clinical benefit
Solid tumor (67)	9	Haploidentical PBMC	CD34+ deleted, CD3+ depleted, CD56+ enrichment	IL-15 + irradiated KT32.A2.41BBL.64	Cryopreserved + culture	≥90%	1–10 × 10^5^ NK cells/kg	5 aGvHD
					9–11 days			

*NSCLC, non-small cell lung cancer; PBMC, peripheral blood mononuclear cell; CRC, colorectal cancer; HCC, hepatocellular carcinoma; CLL, chronic lymphocytic lymphoma; ALL, acute lymphoblastic leukemia; EBV, Epstein–Barr virus; MDS, myelodysplastic syndrome; CML, chronic myelogenous leukemia; RCC, renal cell carcinoma; HD, Hodgkin disease; RMS, rhabdomyosarcoma; MM, multiple myeloma; B-NHL, non-Hodgkin lymphoma*.

In the first phase I clinical trial using the feeder-free *ex vivo* expansion platform, adoptive transfer of NK cells from HLA identical siblings into patients with leukemia or carcinoma was well tolerated and safe alongside *in vivo* NK cell expansion, with only some infusion-related complications (43).

If no HLA identical donor is available, host cells from a receptor–ligand-mismatched donor can be used. If the donor is HLA matched, it is preferentially better if the donor cells are KIR B haplotype. Also, to further improve the outcome, T cell depletion is performed (120). In haploidentical transplantation, at least one KIR ligand is not expressed on the host cells leading to reduced inhibition of donor NK cells. Less inhibited NK cells could lead to better prognosis and might be the best treatment for a good clinical outcome if GvHD can be avoided (38, 121). When haploidentical transplantation is performed, it is strictly necessary to make extensive T cell depletion to avoid GvHD. In most clinical trials, NK cells are collected from leukapheresis followed by a two-step purification procedure, with depletion of CD3^+^ T cells followed by enrichment of CD56^+^ cell (109, 110, 117, 118).

Completed clinical trials with haploidentical donors are safe with only a few reports of infusion-related complications such as dyspnea, nausea, hypertension, stroke, febrile reaction, and vomiting (38, 115). So far, allogeneic NK cell transplantations derived from PBMCs or CD34^+^ cells have shown promising results with engraftment, *in vivo* expansion of NK cells, complete remission, and a 100% 2-year event-free survival in one clinical trial by Rubnitz et al. (109, 112–114, 116).

## Immune Suppression of NK Cells in the Tumor Microenvironment

**Figure 2:**
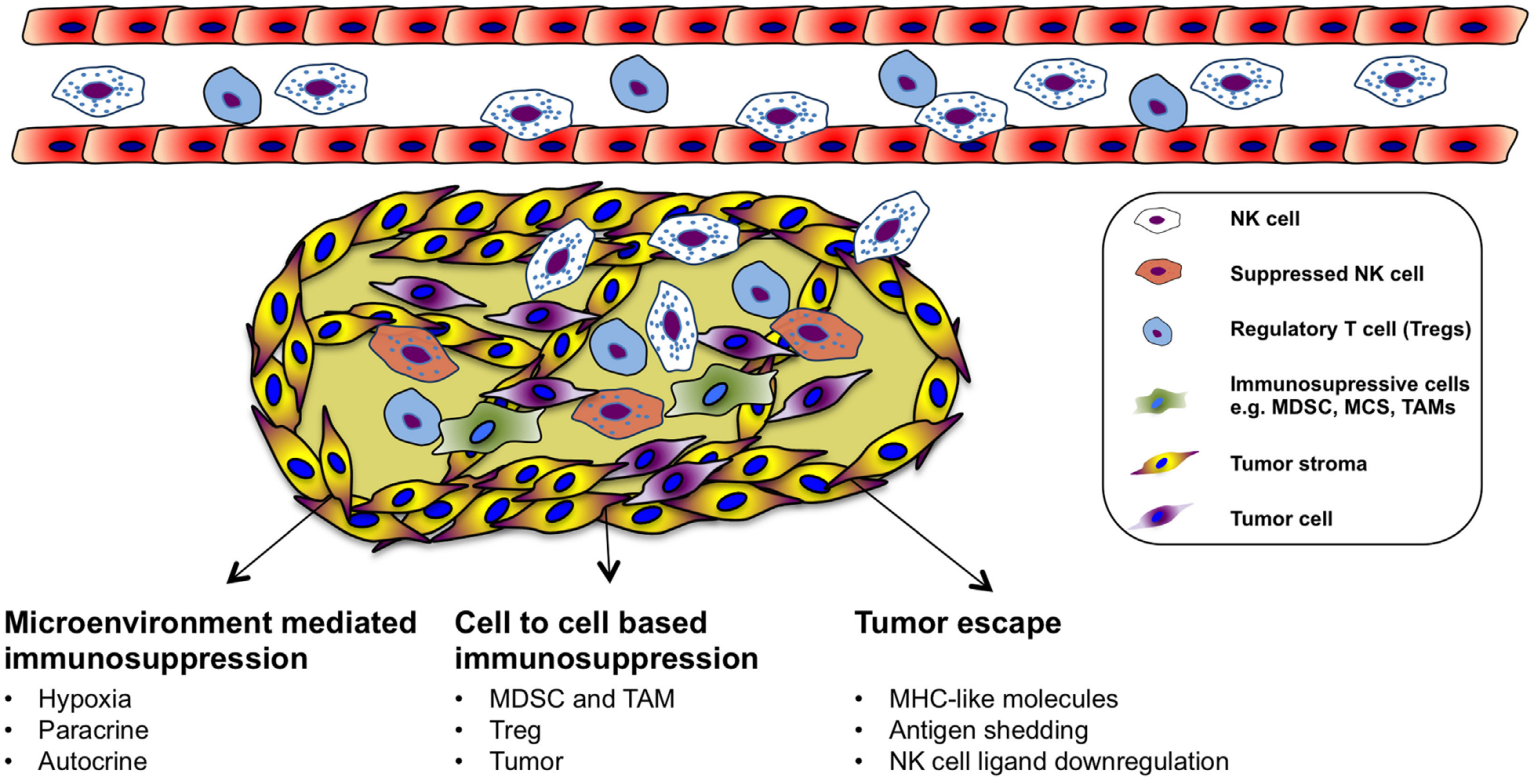
**Immune evasion and immunosuppressive in the tumor microenvironment**.

Natural killer cells can recognize and kill tumor cells *in vitro*. However, their efficiency in targeting solid tumors has not yet been fully acknowledged in the clinical setting even though endogenous and adoptively transferred activated NK cells can be detected in various tumors (122, 123). Nevertheless, not all tumors are equally well infiltrated by NK cells, and many of the infiltrating cells are dysfunctional (124–127). The failure of immune surveillance may in part be due to sustained immunological selection pressure on tumor cells resulting in the development of tumor escape variants that are in fact invisible to the immune system (Figure 2). In addition, cytotoxic function of immune effector cells is also largely suppressed in the tumor microenvironment (128), which could be explained by suppressive tumor-secreted factors as well as suppressive immune compartments, such as myeloid-derived suppressor cells (MDSCs), tumor-associated macrophages (TAM), and regulatory T cells (Figure 2). One of the most studied immune-suppressive cell types associated with tumor progression is regulatory T cells (Treg), characterized by their expression of CD4, high CD25 (CD4^+^CD25^+^CD127^low/neg^) as well as the transcription factor forkhead box P3 (FoxP3) (129). The expansion of Treg population is promoted in different cancers and their accumulation correlates with impaired immune cell function and poor prognosis (130–135). *In vitro*, NK cells are suppressed by Treg cells in a cell contact-dependent manner where membrane-bound TGF-β is utilized to attenuate their cytotoxicity (136). In line with this, inverse correlation between NK cell activity and Treg cell expansion has been observed in patients with gastrointestinal stromal tumor (GIST) (136) as well as in hepatocellular carcinoma patients (137). Treg cells express the high-affinity IL-2 receptor alpha (CD25, IL-2Rα) and need IL-2 for their full function. Recent studies have indicated that NK cell proliferation, accumulation, and activation can be limited by Treg cells through hampering the availability of IL-2 released by activated CD4^+^ T cells (138, 139). Consequently, inadequate IL-2 levels in the tumor microenvironment limits the extent of NK cell-mediated tumor rejection.

Another group of immunosuppressive cells in the tumor is the MDSCs. MDSCs are heterogeneous precursors of the myeloid cells, granulocytes, macrophages, and immature dendritic cells with immunosuppressive activity (140). Recently, MDSCs have been proposed as a key immunoregulator in various solid and hematologic malignancies (141, 142). MDSCs are divided into two groups that can originate from granulocytic (grMDSCs) and monocytic precursors (moMDSCs) (143). In human beings, distinct phenotypes of MDSCs are associated with different types of cancers (144–148). Their suppressive function is mediated by a few different mechanisms such as production of suppressive cytokines including IL-10 and TGF-β, depletion of arginine in the tumor or production of reactive oxygen species (ROS) (144, 149–151). Additionally, recent studies investigated the induction mechanism of MDSCs and how they suppress T cells *in vitro* (152–154). Furthermore, several studies have characterized cytokines that can induce MDSCs from healthy human PBMCs. We found that prostaglandin E2 treated healthy monocytes resemble patient-derived moMDSCs and suppress NK cell responses through TGF-β-dependent mechanism (155). In patients with hepatocellular carcinoma, NK cells were shown to be suppressed by monocytic MDSC in a cell contact-dependent manner, but did not rely on the arginase activity of MDSCs, which is a hallmark function of these cells; however, MDSC-mediated inhibition of NK cell function was revealed to be mainly dependent on the NKp30 on NK cells (146). Moreover, a negative correlation between increased CD33^+^-MDSC accumulation and functional loss of NK cells has been demonstrated in patients with myelodysplastic syndromes (156).

Macrophages are the dominant myeloid-derived population that is found in the tumor microenvironment. TAM has been identified as regulators of solid tumor development based on their capacity to enhance angiogenic, invasive, and metastatic programing of neoplastic tissue (157–160). TAMs could be found in several types of human cancer correlating with poor clinical outcome (161, 162). The immune-suppressive mechanisms applied by TAMs on NK cells in the tumor microenvironment can be different, such as recruitment of Treg, prostaglandin E2-mediated inactivation, and production of IL-10 (163–165). Furthermore, tumors are able to escape NK cells by releasing indoleamine 2,3-dioxygenase and prostaglandin E2, which inhibit the expression of activating receptors of NCRs and NKG2D (166). These molecules are also released by mesenchymal stem cells to inhibit NK cell function in the tumor microenvironment (167). There is a direct association between the surface density of NCRs (NKp46) and the intensity of anti-tumor cytolytic activity of the NK cells (168).

As mentioned earlier, the tumor microenvironment plays a significant role in suppressing NK cell responses against cancer. Therefore, therapies aim to target immunosuppressive cell populations are emerging (169–174). In the next section, some of the alternative ways aiming to enhance tumor-specific targeting and NK cell survival in order to overcome immunosuppressive effect of the tumor microenvironment on NK cells and to improve intra-tumoral NK cell responses will be discussed.

## Future Perspectives

### Genetically Modified NK Cells

**Figure 3:**
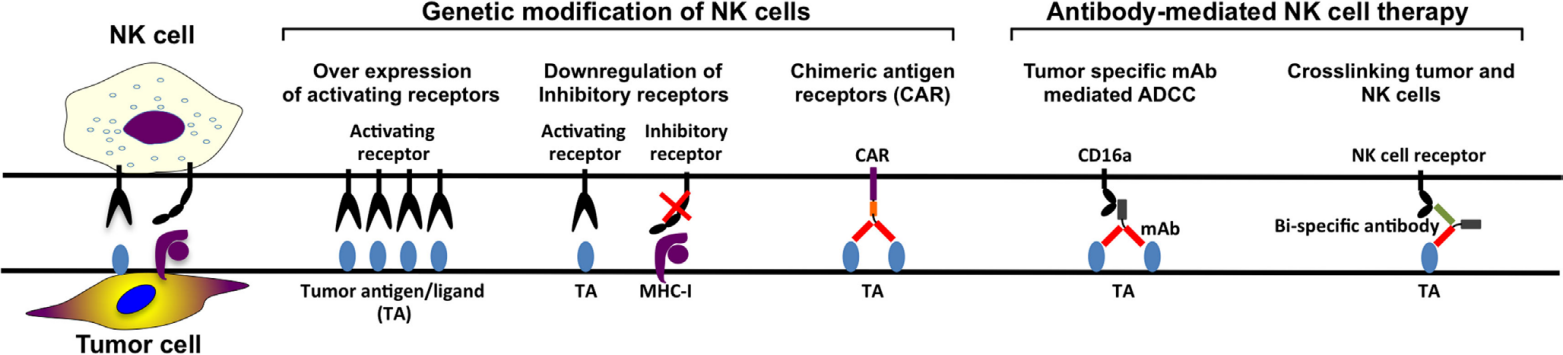
**NK cell therapy approaches**.

In the last decade, several NK cell based anti-cancer products have been taken to clinical trial stage with promising clinical outcomes. However, in order to manufacture more efficient NK cell therapy products, it is essential to develop novel potential strategies such as genetic modification of NK cells (Figure 3). Although NK cells are inherently resistant to retroviral infections (96, 175–177), our group has significantly enhanced retroviral and lentiviral gene delivery to NK cells through enhanced proliferation and targeting intracellular viral defense mechanism by small molecule inhibitors (96). Therefore, it is easier to design genetically modified NK cells expressing cytokine transgenes, silenced inhibitory receptors, overexpressing activating receptors, or retargeting NK cells by expression of CARs on the cell surface. By genetically modifying NK cells to produce cytokines such as IL-2 or IL-15, their survival capacity and proliferation increase and their activation and anti-tumor activity *in vivo* are enhanced (83, 87, 88, 178, 179). To enhance the specificity for the target cells, NK cells can be modified to recognize antigens specifically expressed on the tumor cells.

Furthermore, another approach aiming to enhance tumor specificity is to make use of ADCC. The constant region of the tumor-specific monoclonal antibodies (mAbs) targeting the tumor cells can engage to the FcγRIIIa receptor (CD16a) on the NK cell, activating the NK cell. However, NK-92 cell line cannot perform ADCC since they lack CD16a expression (80, 85). This defect on NK-92 cells can be reverted by the introduction of CD16a through genetic modification so that they are able to perform ADCC in antibody combination treatments (180). Finally, CAR-modified NK cell lines can also function as tumor-specific standardized and characterized NK cell-based therapy products. Most of the NK cell lines require further *in vivo* characterization with a potential to become standard NK cell-based products for certain tumors.

### Monoclonal Antibodies

When the antigen-binding fraction (Fab) of the antibody binds to the tumor target cell and the constant region (Fc) of the antibody binds to CD16 on the NK cells, NK cells get activated and ADCC is triggered. Several different mAbs have been developed for targeting specific tumor antigens, such as anti-CD20 (retuximab), anti-Her2 (trastuzumab), anti-CD52 (alemtuzumab), anti-EGFR (certuximab), and anti-CD38 (daratumumab) (181). Daratumumab treatment of patients with relapsed myeloma has mild infusion-related reactivity, complete or very good partial responses with reduced bone marrow plasma cell levels (182). mAbs bind to the target tumor cell plus engaging CD16 on NK cells and other cell types resulting in killing of tumor cell by ADCC both *in vivo* and *in vitro* [reviewed in Ref. (183)]. New generations of mAbs have been developed to increase ADCC and complement-dependent cytotoxicity. Second-generation anti-CD20 mAbs, such as veltuzumab (hA20) (184, 185) and ofatumumab (HuMax-CD20) (186–193), have the advantage of being humanized or of fully human origin. Both veltuzumab and ofatumumab had promising preliminary outcomes in various studies (184, 186, 187, 189, 190, 193). The benefit of third-generation anti-CD20 mAbs, ublituximab (TG-1101), ocaratuzumab (AME-133) (194, 195), and obinutuzumab (GA-101) (196–200), is that they are both humanized and that their Fc regions have been modified for increased binding affinity to CD16a. So far, the most studied third-generation anti-CD20 mAbs is obinutuzumab. The overall response rate for obinutuzumab is 44.6%, which is higher than the overall response rate for rituximab treatment which is 33.7% (200). In the same study, the progression-free survival did not promote obinutuzumab over rituximab. By increased affinity between CD16a and mAb better NK cell cytolysis can be induced by ADCC. Ublituximab, ocaratuzumab, or obinutuzumab-treated NK cells from CLL patients or healthy donors have more efficient ADCC compared to same cells treated with first- or second-generation anti-CD20 mAb *in vitro* (201–203).

Monoclonal antibody therapies in combination with already existing treatments can potentially enhance NK cell activity in anti-tumor therapy. The completely human IgG4 anti-KIR antibody, IPH-2102, has been tested in several clinical trials for hematological diseases both as single treatment and as combination (204, 205). Some clinical trials for combination treatment of advanced solid tumors with anti-KIR antibodies are done as well, for example, in combination with anti-CTLA antibody or anti-PD1 antibody (NCT01750580 and NCT01714739, respectively). Thus, use of mAbs enhancing ADCC and stimulation of NK cells as well as blocking NK cell inhibition could potentially improve outcome of clinical anti-cancer NK cell products (Figure 3).

### Bi- and Trispecific Antibodies

Likewise designing CARs through tumor-specific mAbs can be used to engineer bi- and trispecific antibodies crosslinking CD16 with tumor-specific mAbs in order to enhance NK cell tumor reactivity (Figure 3). Briefly, the design of bi- and trispecific antibodies, fusing the Fab region of the antibody targeting the tumor cell antigen, such as CD19, CD20, and CD33, in combination with another Fab region recognizing CD16 on NK cell leads to stimulation of the NK cells followed by tumor cell killing. This technology makes it possible to select the amount of NK cells that should be activated as well as it is possible to add more Fab regions targeting other tumor-associated antigens. These Fab regions can be exchanged to other tumor-associated antigen-recognizing antibody parts, as long as the part crosslinking CD16 on the NK cell is present (206, 207).

### Chimeric Antigen Receptors (CARs)

Design of CARs using antigen-specific variable part of these tumor antigen antibodies fused with intracellular lymphocyte stimulatory molecules (CD3ξ, CD28, 4-1BB) enables high-affinity specific recognition of tumor antigens and tumors. CAR modifications of T cells have been studied extensively and have led to several phase I and phase II clinical trials (208–211). NK cells are less explored and so far only two clinical trials using CAR NK cells have been approved. The first study (NCT00995137) at St. Jude Children’s Research Hospital is completed and was a phase I clinical trial with 14 relapsed or refractory B-lineage ALL patients below 18 years. Haploidentical NK cells were expanded by co-culture with irradiated K562 cell line expressing IL-15 and 4-1BB ligand on the surface to be transduced with a signaling receptor binding CD19 (anti-CD19 CAR). The second study (NCT01974479) is a phase II pilot study, which is still recruiting refractory B-lineage ALL patients in all ages. NK cells are expanded by co-culture with K562 cells as the previous trial, together with IL-2 before transduction with the same construct. The patients will also receive IL-2 after NK cell administration to support NK cell viability and expansion. Although CAR T cell studies have been extremely promising, CARs designed for T cell therapies are still suboptimal for NK cells. Thus, it is essential to further optimize the construct design, especially the intracellular stimulatory adapter molecules, in order to trigger most efficient NK cell responses.

### Immunomodulatory Drugs (IMiDs)

Immunomodulatory drugs (IMiDs) such as thalidomide, lenalidomide, and pomalidomide, can stimulate both NK cells and T cells, potentially resulting in better targeting cancer cells (212). Lenalidomide upregulates TRAIL molecules on NK cells and enhances anti-tumor activity (14, 15). So far, several different malignancies, both solid and hematological, have been treated with IMiDs. A large part of the nearly 100 clinical trials with IMiDs that has been reported with results to clinicaltrials.gov is treatment of myeloma, lymphoma, and leukemia. IMiDs can be used as combination treatment, such as lenalidomide in combination with IPH-2102, anti-inhibitory KIR antibody therapy (205). Lenalidomide expands and activates the NK cells, while anti-inhibitory KIR antibody (IPH2101) promotes NK cell recognition and lysis of tumor cells. This combination could give a better therapeutic outcome.

### Combination Treatments

It is possible that NK cell products cannot fully eliminate tumor cells due to several immunosuppressive effects of tumor microenvironment as well as reduced *in vivo* expansion and cytotoxicity. These obstacles could be overcome by combination treatments using NK cell therapy products together with other drugs either directly targeting tumor cells or modulating cytotoxic activity of NK cells. As mentioned earlier, use of mAbs and IMiDs together with appropriate NK cell products could enhance tumor targeting and elimination. Another way to enhance NK cell-mediated killing is to combine drug therapy with NK cell stimulating cytokines such as IL-2, IL-12, IL-15, and IL-21 (213).

Furthermore, chemotherapy in combination with NK cell infusions is an alternative way to overcome tumor-induced dysfunctions. NK cells from haploidentical donor require combination treatments with the intense chemotherapy drugs high-dose fludarabin and cyclophosphamide (Hi-Cy/Flu) plus daily infusion of IL-2 to be able to expand *in vivo* (38). Total body irradiation could help to create immunological space for expanding NK cells in addition to chemotherapy after short-term *ex vivo* activation of NK cells (214).

## Conclusion

In this review, we have summarized current NK cell-based therapy strategies as well as some of the challenges that need to be addressed. Even though NK cell-based therapies represent one of the most promising strategies to combat cancer, to our knowledge, no clinical trial has clearly demonstrated a significant benefit in patients with malignancies. This is in part due to the lack of prospective large-scale clinical trials and partly due to a lack of consensus in which NK cell product preparation would show the best effect. Further comparative clinical studies are definitely warranted; however, the design of such clinical trials is challenging due to the advanced therapy regulations in major countries such as European Union member states and the United States of America. Although cell therapy clinical trials are reaching a log-linear expansion, the number of NK cell-based therapies is not aligned with this increase. Nevertheless, there is a lot of promise in early clinical and pre-clinical data that cannot be omitted. In the near future, different NK cell-based products will reach multicenter clinical trial stage and we will start to see efficacy data.

Separately, NK cell-based therapies are in theory complementary to many different upfront, maintenance, and late-line therapies. Further studies clarifying the complementary efficacies and synergies have to be initiated to conclusively state if there is any place for these intriguing cells in search for an effective treatment of cancer.

## Author Contributions

All the authors performed the review of the literature, wrote, and edited the manuscript.

## Conflict of Interest Statement

The authors declare that the research was conducted in the absence of any commercial or financial relationships that could be construed as a potential conflict of interest.
